# Are joint and soft tissue injections painful? Results of a national French cross-sectional study of procedural pain in rheumatological practice

**DOI:** 10.1186/1471-2474-11-16

**Published:** 2010-01-25

**Authors:** Serge Perrot, Françoise Laroche, Coralie Poncet, Pierre Marie, Catherine Payen-Champenois

**Affiliations:** 1 Service de Médecine Interne et Thérapeutique, Hôpital Hôtel Dieu, Université Paris Descartes, Unité INSERM U987, Paris, France; 2 Centre d'évaluation et de traitement de la Douleur, Hôpital St Antoine, Paris, France; 3 DOCS International, Issy les Moulineaux, France; 4 BMS, Rueil-Malmaison, France

## Abstract

**Background:**

Joint, spinal and soft tissue injections are commonly performed by rheumatologists in their daily practice. Contrary to other procedures, e.g. performed in pediatric care, little is known about the frequency, the intensity and the management of procedural pain observed in osteo-articular injections in daily practice.

**Methods:**

This observational, prospective, national study was carried out among a French national representative database of primary rheumatologists to evaluate the prevalence and intensity of pain caused by intra-and peri-articular injections, synovial fluid aspirations, soft tissue injections, and spinal injections. For each physician, data were collected over 1 month, for up to 40 consecutive patients (>18-years-old) for whom a synovial fluid aspiration, an intra or peri-articular injection or a spinal injection were carried out during consultations. Statistical analysis was carried out in order to compare patients who had suffered from pain whilst undergoing the procedure to those who had not. Explanatory analyses were conducted by stepwise logistic regression with the characteristics of the patients to explain the existence of pain.

**Results:**

Data were analysed for 8446 patients (64% female, mean age 62 ± 14 years) recruited by 240 physicians. The predominant sites injected were the knee (45.5%) and spine (19.1%). Over 80% of patients experienced procedural pain which was most common in the small joints (42%) and spine (32%) Pain was severe in 5.3% of patients, moderate in 26.6%, mild in 49.8%, and absent in 18.3%. Pain was significantly more intense in patients with severe pain linked to their underlying pathology and for procedures performed in small joints.

Preventative or post-procedure analgesia was rarely given, only to 5.7% and 36.3% of patients, respectively. Preventative analgesia was more frequently prescribed in patients with more severe procedural pain.

**Conclusion:**

Most patients undergoing intra-or peri-articular injections, synovial fluid aspirations and spine injections suffer from procedural pain. Most patients experience usually mild procedural pain and procedural pain management is uncommonly provided by physicians. Specific research and guidelines for the management of procedural pain related to rheumatologic care should be established to improve the quality of care provided by physicians.

## Background

Patients undergoing physical examination, diagnostic and therapeutic interventions are often subject to pain as a result of their medical care. Despite this, nearly two-thirds of patients undergoing potentially painful medical procedures do not receive any form of analgesia [1]. Management of care-related pain is now a priority in paediatric care where lots of review and guidelines have been published [2] but remains poorly studied in adults, and particularly in rheumatological practice.

Pain induced by medical procedures i.e. "procedural pain" has largely been studied in paediatric populations [3,4] and, with the exception of pain linked to a few specific procedures such as turning, drain removal and catheter insertion [5-8], there is little information concerning procedural pain in adults. A few studies have investigated procedural pain in surgical or oncology patients [9-12], but there are no data concerning the intensity or frequency of pain induced by medical procedures in rheumatological conditions. Invasive procedures such as punctures and infiltrations are carried out routinely in the diagnosis and therapy of musculoskeletal disorders, not only by rheumatologists, but also by family physician [13]. However, it has not been determined whether these procedures are painful to patients. Although Wang et al. reported that injection of corticosteroids into the elbow did not increase pain these authors did not investigate specifically pain induced by the procedure itself [14].

Since invasive procedures such as corticosteroid injections, anaesthetic injections, and dry needling are carried out frequently in rheumatology [15,16], it is important to better understand which factors influence procedural pain, and how it can be managed effectively. Some procedures for the management of induced pain have been established in rheumatology department, but not validated and published, and most approaches to date have used empirical treatment. The results of studies on the use of topical lidocaine-prilocaine cream (EMLA) before vascular puncture and corticosteroid injections are contradictory [17-19]. It therefore seemed pertinent to evaluate pain linked to different procedures in rheumatology, and how this pain is currently managed.

This national observational prospective study was set up to evaluate the frequency of puncture and/or infiltration-induced pain in rheumatological practice, and the different preventative and post-procedure therapeutic approaches currently used to manage procedural pain.

## Methods

### Study design

This was an epidemiological observational, cross-sectional, national study carried out among French rheumatologists between 2006 and 2007, after obtaining **approval of french national ethical committee (CCTIRS)**. Each participating rheumatologist was asked to record data for all consecutive adult patients who underwent a diagnostic or therapeutic procedure (puncture, or infiltration, or both) at the time of consultation, irrespective of localisation. The observation period extended over 1 month of visits.

### Selection of rheumatologists

Rheumatologists were selected from the French rheumatologist' representative TVF database (source CEGEDIM group). In order to guarantee a high level of respondents, details of the study and a request to participate were sent out to all rheumatologists across France in two mail-shots. This enabled some adjustment in the second mailing according to the level and regionality of the response to the first. The rheumatologists in the first mail-shot were chosen by drawing lots stratified according to administrative region.

The experience of each rheumatologist was determined indirectly from their age, assuming that they had started practising professionally when they were 27-years-old. The study was proposed to 1800 French rheumatologists with private practice. After 2 contacts and propositions, 339 rheumatologists accepted to participate. Finally, of these 339 rheumatologists, only 252 rheumatologists have participated actively in the study and have recruited patients. The demographics of these rheumatologists were compared to the global demographics of French rheumatologists and did not demonstrate significant difference, according to age, sex-ratio, number of years of practicing, location.

### Patient selection

All consecutive adult patients (>18 years of age) suffering from a non-malignant musculoskeletal pathology for which a diagnostic and/or therapeutic procedure (puncture, or infiltration, or both) was carried out at the time of consultation were recruited (maximum of 40 patients per rheumatologist). As this was an observational study, the only exclusion criteria were mesotherapy or acupuncture procedures. All patients provided informed consent before procedure.

### Data collection

The following data were recorded for each patient: age; sex; underlying pathology for which the procedure was required; previous experience of the patient regarding a similar type of procedure; details of the procedure [20] performed (type, localisation, aim, planned or not); analgesic protocols (preventative, post-procedure) used; evaluation of pain before procedure and during procedure, and of the patients' experience post-procedure.

### Evaluation of pain intensity

Pain intensity was evaluated using verbal scale (none, mild, moderate or severe). The verbal rating scale is a commonly used scale, easier to understand than Visual Analog Scale. It has been validated in many studies and is one of the three most commonly used scale [21].

Patient's pain intensity was assessed immediately before the procedure, considering mean pain intensity during the previous week before procedure. Then, after the procedure, patients had to report to the physician their level of pain induced by the procedure,.

### Primary evaluation criteria

The primary objectives of the study were to evaluate: (i) the respective frequency of different articular and peri-articula	r procedures in rheumatological primary practice; (ii) the frequency and intensity of pain linked to these different procedures; (iii) the demographic factors for procedural pain. (iv) the type and frequency of analgesic treatment (preventative and post-procedural) administered for procedural pain;

### Statistical analysis

Statistical analysis was carried out in order to compare patients who had suffered from pain whilst undergoing the procedure to those who had not. Explanatory analyses were conducted with the characteristics of the patients to explain the existence of pain. When patients had more than one procedure during the visit, each procedure was assessed separately.

For descriptive data, quantitative variables are presented as number (n), means and standard deviation (SD), and qualitative variables as n and % of available data. For the descriptive and univariate analysis, quantitative variables were analysed using the Wilcoxon or Mann-Whitney test, and qualitative variables were compared using the Chi^2^-Pearson or Fisher's exact test. The level of significance selected was 0.05.

Multivariate analysis was carried out by stepwise logistic regression with the dependent variable being the presence or absence of pain. Significant factors to the threshold of 20% in univariate analysis were included in multivariate analysis. Entry threshold was set-up at 10% and conservation threshold to 15%.

Statistical analysis was carried out using SAS^® ^(version 8.2; SAS Institute, North Carolina, USA).

## Results

### Rheumatologists

A total of 240 French rheumatologists took part in this study (57.7% male, mean age 49 years). 68.1% had exclusive private practice, 31.4% had a mixed hospital/surgery practice and the rest were practicing exclusively in hospital. The rheumatologists each had an average of 22 years experience.

### Patient characteristics

Data were analysed for 8446 patients who underwent a puncture, or infiltration, or both, during visit to 240 rheumatologists across France. The mean age of the patients was 62 ± 14 years and the majority were female (64.4%). The demographic characteristics of the patients are summarised in Table 1.

**Table 1:** Demographic and clinical characteristics of the patients.

		(n = 8446)
Age (mean ± SD), years		62 ± 14
<50		1613 (19.2%)
50-59		1860 (22.0%)
60-69		1945 (23.1%)
70-79		2050 (24.3%)
≥80		970 (11.5%)
Sex (female/male), %		64/36
Number of patients taking analgesic medication for their underlying pathology during the previous week		5643 (67%)
Pain linked to the underlying pathology during the previous week (n = 8434)	no pain, n (%)	78 (0.9%)
	mild pain, n (%)	1179 (14.0%)
	moderate pain, n (%)	4067 (48.2%)
	severe pain, n (%)	3110 (36.9%)

*Data missing for some patients.

In 31.3% of the cases patients had joint osteoarthritis, tendonitis in 18.1%, radiculalgia in 9.1%, back pain in 7.2%, inflammatory rheumatological disorder in 3.8% (mostly rheumatoid arthritis), and a tunnel syndrome in 4.4%. In 66.8% of the cases, patients were prescribed analgesics for the pain related to the underlying pathology.

### Types of procedure performed

In total, 91.8% of patients underwent only one procedure. The knee was the main site of intervention (45.4%), followed by spine (19.1%), shoulder (15.0%) and small joints (12.0%). The procedure was unilateral in 91.6% of patients, intra-articular in 57.0%.

The procedure was carried out for therapeutic purposes in the majority of patients (91.8%), and was diagnostic and therapeutic in 6.8%. Over half of the patients (56.1%) were given a corticosteroid injection, while 26.0% received an injection of hyaluronic acid. In the remaining cases, procedure was a synovial fluid aspiration or combination of aspiration and injections (Table 2). Approximately half (53.9%) of the interventions were planned before the consultation.

**Table 2:** Type of procedure performed during the visit

	n = 8123
Steroid injection	4557 (56.1%)
Hyaluronic acid injection	2108 (26.0%)
Joint aspiration and steroid injection	555 (6.8%)
Steroid and analgesic drug injection	271 (3.3%)
Joint aspiration and hyaluronic acid injection	223 (2.8%)
Joint aspiration alone	133 (1.6%)
Other procedures	276 (3.4%)

### Incidence of procedural pain

**Figure 1:**
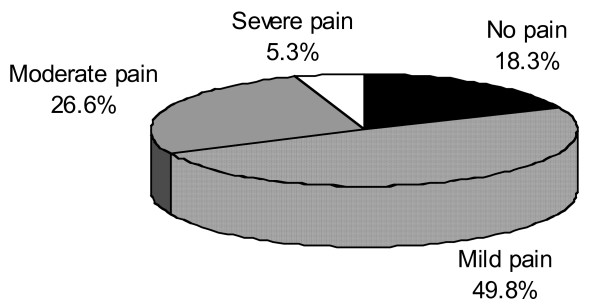
**Global pain intensity linked to all the procedures performed (n = 8393)**.

The majority of patients (81.7%) experienced some degree of pain induced by the procedure. Pain was considered to be severe by 5.3% of patients, moderate by 26.6%, and mild by 49.8%. A small proportion of patients (18.3%) experienced no pain (Figure 1).

### Patient characteristics linked to procedural pain

**Figure 2:**
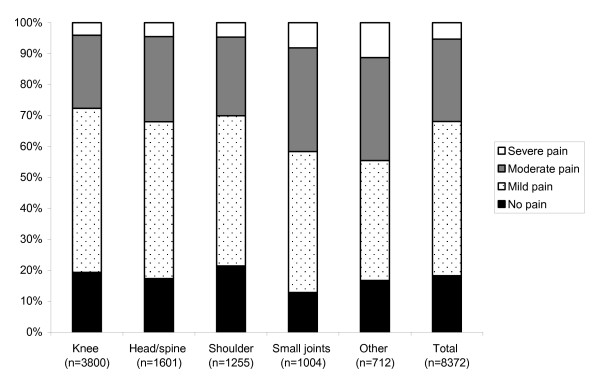
**Intensity of procedural pain according to site of intervention (n = 8372): percentage of each verbal rating category for each procedure site**.

Univariate analysis demonstrated that the intensity of pain varied significantly associated with the site of the injection/aspiration. Pain was considered to be more intense (moderate to severe) in 41% of cases involving the small joints, 32% involving the head and spine, 30% for the shoulders and 28% for the knees (Figure 2). Other factors like age (p < 0.001), sex of the patient (p < 0.001), type of underlying rheumatological pathology (p < 0.001), pain in the previous week related to this underlying pathology (p < 0.001), analgesic treatment for pain related to this underlying pathology (p < 0.001), procedure planned before the visit (p = 0.001) and patient's procedural perception evaluated by the physician (p < 0.001) were significant factors associated with procedural pain.

On the contrary, univariate analysis demonstrated that actual flare of the disease, history of previous procedure, number of procedures performed on the same visit, body-side of the procedure, type of injection (intra vs extra-articular), aim of the procedure (therapeutic vs diagnostic procedure) were not significant factors for the occurrence of procedural pain.

From this univariate analysis, fourteen demographic and pain ratings explanatory variables linked to the occurrence of pain during the procedure (with significant threshold of 20% in univariate analysis) were included in the multivariate analysis (Table 3). It demonstrated significant differences for intensity of pain linked to the underlying pathology during the week preceding the procedure (p < 0.0001), site on which the intervention was required (p = 0.0006) and the patient's procedural perception evaluated by the physician (p < 0.0001). Thus, pain was considered to be significantly and independently more intense in patients with more severe pain linked to their underlying pathology, for procedures performed in small joints, globally in accordance with the perception of the procedure by the rheumatologists. There was a trend towards more intense pain in women, but not significant. The use of preventative analgesic protocol was also significantly associated with the presence of procedural pain in univariate analysis (p = 0.005), demonstrating that physicians were taking into account the possibility of that induced pain.

**Table 3:** Multivariate analysis of existence of pain versus no pain after the procedure

Explanatory variables	OR *	95% CI	P-value**
**Patient perception of the procedure assessed by the physician**			< 0.0001
Very/Quite badly ^R^	1		
Quite well	0.271	[0.085 - 0.865] (S)	
Very well	0.013	[0.004 - 0.041] (S)	

**Pain intensity related to underlying pathology in the previous week**			
Absent ^R^	1		< 0.0001
Mild	1.348	[0.780 - 2.330] (NS)	
Moderate	1.780	[1.039 - 3.051] (S)	
Severe	2.024	[1.165 - 3.513] (S)	

**Underlying pathology**			0.0006
Acute ^R^	1		
Chronic	1.725	[1.099 - 2.708] (S)	
Inflammatory rheumatological disorder	2.521	[1.474 - 4.310] (S)	
Joint osteoarthritis	2.302	[1.548 - 3.423] (S)	
Flare-up over degenerative pathology	1.972	[1.301 - 2.988] (S)	
Post-traumatic	2.967	[1.483 - 5.935] (S)	
Back pain	1.755	[1.077 - 2.861] (S)	
Radiculalgia	1.394	[0.856 - 2.272] (NS)	
Tunnel syndrome	1.975	[1.148 - 3.399] (S)	
Tendonitis	1.310	[0.876 - 1.960] (NS)	
Bursitis	1.241	[0.716 - 2.151] (NS)	
Cristal arthropathy	1.883	[0.772 - 4.595] (NS)	

**Sex**			
Male ^R^	1		0.0557
Female	1.141	[0.998 - 1.304] (NS)	

**Site of procedure**			
Knee ^R^	1		0.0889
Spine	1.160	[0.854 - 1.576] (NS)	
Shoulder	1.174	[0.901 - 1.530] (NS)	
Small joints	1.551	[1.137 - 2.118] (S)	
Others	1.212	[0.903 - 1.627] (NS)	

Multivariate analysis was carried out with variables selected from univariate analysis that demonstrated a significant threshold of 20%.

Multivariate analysis was performed on 7518/8446 patients. 928 observations were not included due to missing data.

*OR: Odds-Ratio

CI: Confident Interval

** Chi^2 ^test significance

S: significant CI

NS: non significant

^R^: Reference.

### Perception of rheumatologists regarding procedural pain experienced by their patients

**Figure 3:**
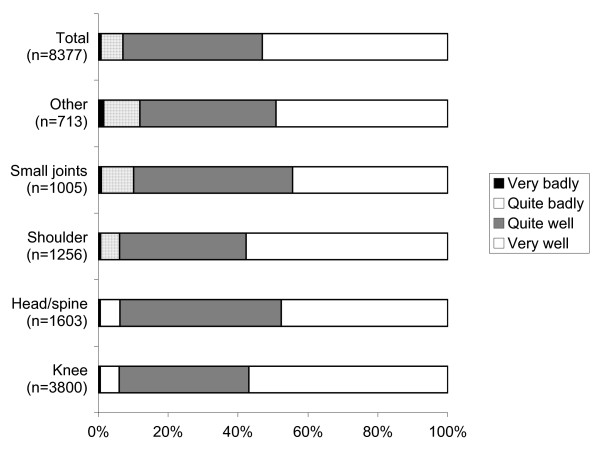
**Perception of patient's procedural pain by rheumatologists**. (n = 8377): percentage of each verbal category for each procedure site.

On the whole, rheumatologists thought that the majority of their patients had tolerated the procedure very (53.1%) or quite (39.8%) well, and that only 0.7% of patients had suffered badly during the procedure (Figure 3). This was in accordance with the intensity of pain experienced during the procedure by their patients (kappa score: 0.44).

### Management of procedural pain

Less than half of the patients received any analgesia (either preventative or curative) for their pain. Only 5.7% of patients were given preventative analgesic medication, usually local anaesthesia (34.8%), xylocaïne patches (20.5%), acetaminophen alone (14.3%; Step 1 analgesics I) and Step 2 analgesics (11.2%). In most patients this was initiated by the physician (82.6% of cases) rather than at the request of the patient. The mean duration between receiving preventative analgesia and the procedure was 38 ± 74 min. There was no significant difference in the use of preventative analgesia between patients undergoing a single procedure and those undergoing two (p = 0.683).

Post-procedure analgesia was given to 36.3% of patients and was initiated by the physician in 94.6% of the cases. The most common analgesics prescribed were step 2 analgesics (weak opioids combined to acetaminophen) (35.8%), non-steroidal anti-inflammatory drugs (NSAIDs) (34.8%) and acetaminophen alone (34.2%).

## Discussion

This research highlights a major problem in rheumatology care. Pain caused by injections and aspirations from the joints is a common occurrence for patients, in 81.7% of the cases and may represent a significant medical stressor. The large sample size of our study increases the relevance of the findings and the public health significance of the problem. These results imply that more should be done to manage the pain caused by procedures to relieve suffering and functional impairments in osteo-articular pathological conditions. Procedural pain has been investigated previously in a number of hospital populations [1-4,6-13,22], mostly in cancer management, but never in patients with painful musculoskeletal conditions, in rheumatological practice.

Our observational study shows that the procedural pain can be moderate to severe in intensity in approximately one-third (31.9%) of patients, particularly if the procedure involves the small joints. Preventative and post-procedural pain management, both by pharmacological and non-pharmacological approaches should be performed specifically in these situations. There is also evidence that pain is more intense in patients with severe pain linked to their underlying pathology, and this also suggests that better pain control of the underlying pathology may decrease the discomfort related to the procedure.

Local infiltration therapies including corticosteroid injections are commonly used in the management of musculoskeletal disorders and have been shown to bring about short-term pain relief in a number of conditions including osteoarthritis, inflammatory arthritis, synovitis, tendonitis and bursitis [23,24]. In the current study, over half of our patients received a corticosteroid injection during the visit and a further 25% received an injection of hyaluronic acid. While it was previously reported that the injection of corticosteroids into the elbow did not increase pain linked to the underlying pathology [14], we have demonstrated that infiltrations themselves can be extremely painful in some people. Many medical and non-medical staff constantly under-estimate pain in their patients [23], or are unaware that some medical procedures can cause pain [22]. This is borne out by the results of a study by Puntillo et al. where almost two-thirds of patients undergoing six specific medical procedures did not receive any analgesic or opiate medication [1] and this in part explains their reluctance to prescribe preventative analgesic medication. Several studies have reported that vascular procedures, including venipuncture and arterial puncture, are a significant cause of pain, and there is some evidence that application of anaesthetic lidocaine-prilocaine cream (EMLA) to the puncture site before the procedure can successfully prevent pain [18,19]. Despite evidence of efficacy however, local anaesthetic creams are still rarely used routinely in hospitals [3,9]. However, perception by rheumatologists of the conditions of the procedure was well correlated with the occurrence of procedure-related pain, and with the prescription of preventative analgesia, even if this doesn't demonstrate a real efficacy to prevent procedure-related pain.

The results of this study are subject to some limitations. Recruitment bias was overcome by selecting rheumatologists from a national list. Self-selection bias of patients is difficult to determine because the number and characteristics of non-volunteers were not assessed: rheumatologists were invited to include all consecutive patients, within a month, that needed joint procedure. Since this was an observational study, and because we did not want to burden the assessment procedure and the protocol for participating rheumatologists, we did not ask rheumatologists to assess patients who do not accept to participate in the study. Also for practical reasons and to raise the number of physicians and patients involved, we have used the more simple method to assess pain, a categorical verbal rating, that is a validated but a less accurate method than pain Visual Analog Scale (VAS) or pain Numerical Rating Scale (NRS). It is obvious that a pain VAS would have been an improvement. The study highlighted some demographic correlates of pain but it did not include any psychological or behavioral variables, as anxiety or fear of the procedure, that could be interesting data to analyse. Another limitation of our study is that we did not differentiate the devices used by the physicians. Several authors have emphasized the fact that size of the syringe and needle may influence pain and tissue trauma during procedure. Recent findings have also pointed out the usefulness of sonographic-guided injections to improve the quality of care in rheumatological procedures [25]. The last limitation concerning our study is that we did not precisely assess the levels of experience of the recruiting physicians, as physician training and number of injections performed regularly are important factors that may influence physicians confidence and quality of care [26]. However, in our study, all physicians were rheumatologists, with a mean experience in joint and soft tissue injections of 22 years.

## Conclusion

It is important to make rheumatologists more aware that patients may find infiltrations and/or joint aspiration painful so that specific analgesic protocols can be implemented. In this study, analgesia was usually initiated by the rheumatologist rather than at the request of the patient. Thus, physicians have a pivotal role to play in the management of pain linked to musculoskeletal disorders and to any interventional procedures performed. Greater prescription of preventative and post-procedural analgesic procedures (both pharmacological and non-pharmacological) should be encouraged in order to improve the quality of care offered by rheumatologists.

## Competing interests

This study was performed with the support of an independent grant provided by BMS-France pharmaceutical company.

## Authors' contributions

SP conceived of the study, participated in its design and drafted the manuscript, FL participated in the design of the protocol and in its writing, CP performed the statistical analysis, NS participated in the design of the protocol and in coordination, CPC participated in the design of the study and in its coordination. All authors read and approved the final manuscript.

## Pre-publication history

The pre-publication history for this paper can be accessed here:

http://www.biomedcentral.com/1471-2474/11/16/prepub
